# Supplementary material for: The interactome of intact mitochondria by cross-linking mass spectrometry provides evidence for coexisting respiratory supercomplexes
Source: Mol Cell Proteomics. 2017 Dec 8;17(2):216–32. doi: 10.1074/mcp.RA117.000470 (PMC5795388; doi:10.1074/mcp.RA117.000470)
Supplement: Supplemental Data [file supp_RA117.000470_133922_0_supp_23978_fzffwf.zip › spectra_annotation/mito_spectra_annotation/8-1-5-1-4-1.pdf]

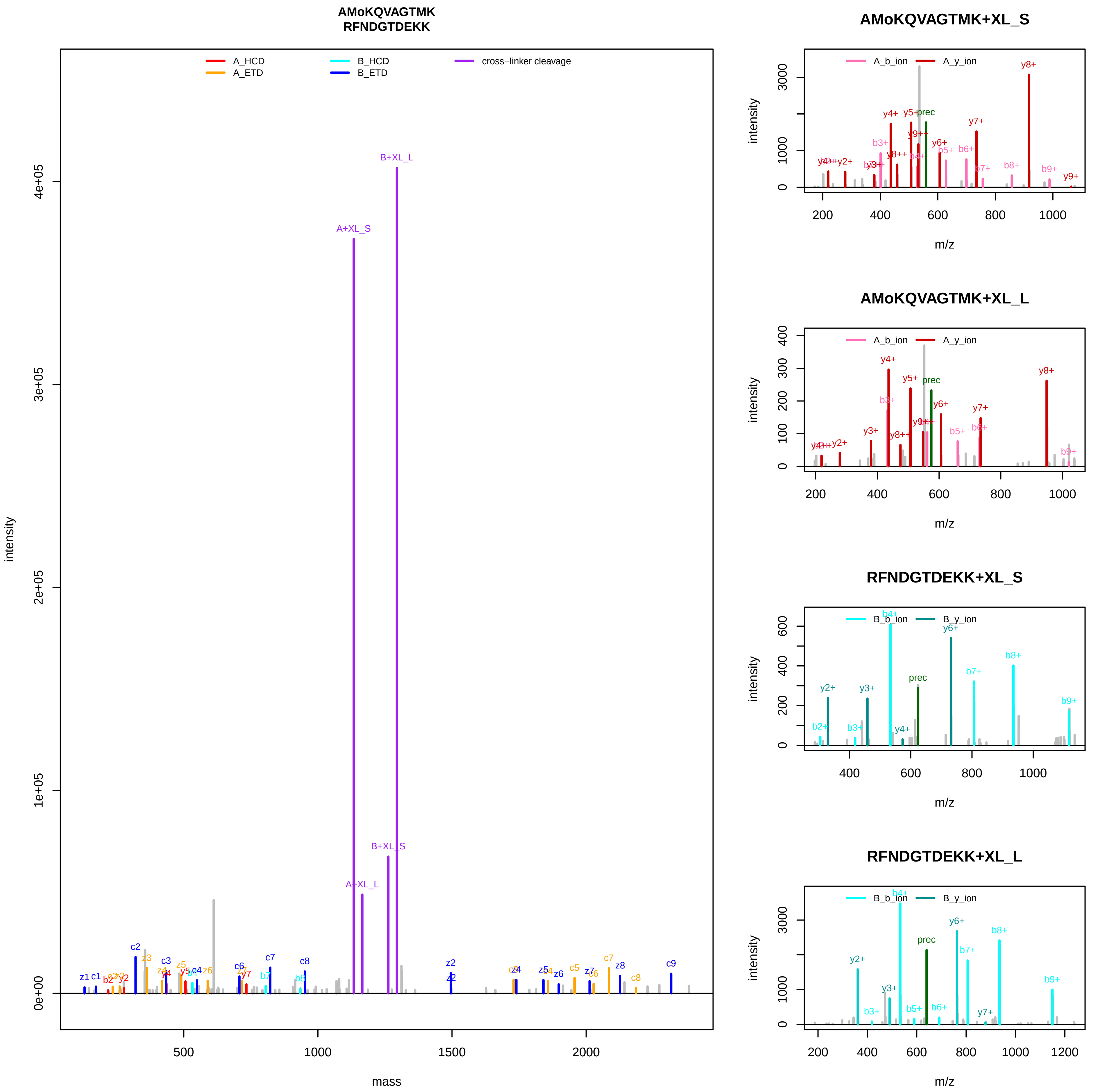

AMoKQVAGTMK
RFNDGTDEKK
A_HCD
A_ETD
B_HCD
B_ETD
cross−linker cleavage
B+XL_L
A+XL_S
B+XL_S
A+XL_L
intensity
mass
0e+00
1e+05
2e+05
3e+05
4e+05
500
1000
1500
2000
c2
c3
c4
c6
c7
c8
c9
z1
c1
b2
z2
z3
z5
z6
z7
z8
y2
y4
y5
y7
b7
b8
c5
c8
AMoKQVAGTMK+XL_S
AMoKQVAGTMK+XL_L
RFNDGTDEKK+XL_S
RFNDGTDEKK+XL_L
A_b_ion
A_y_ion
B_b_ion
B_y_ion
prec
m/z
y8+
y7+
y6+
y5+
y4+
y3+
y2+
y9+
y8++
y9++
y4++
b3+
b4+
b5+
b6+
b7+
b8+
b9+
b2+
